# A model for evaluation of the electric activity and oxygenation in the erector spinae muscle during isometric loading adapted for spine patients

**DOI:** 10.1186/s13018-020-01652-3

**Published:** 2020-04-17

**Authors:** Lars Ekström, Qiuxia Zhang, Josefin Abrahamson, Joel Beck, Christer Johansson, Olof Westin, Carl Todd, Adad Baranto

**Affiliations:** 1 Institute of Clinical Sciences, Department of Orthopedics, Sahlgrenska Academy, University of Gothenburg and Sahlgrenska University Hospital, R-House, Floor 7, SE-431 80 Mölndal, Gothenburg, Sweden; 2 The Carl Todd Clinic, 5 Pickwick Park, Park Lane, Corsham, SN13 0HN UK

**Keywords:** Spinal stenosis, EMG, NIRS, Paraspinal muscle, Muscle oxygenation, Muscle activity, Laminectomy

## Abstract

**Background:**

Simultaneous measurement of electromyography (EMG) and local muscle oxygenation is proposed in an isometric loading model adjusted for patients that have undergone spinal surgery.

**Methods:**

Twelve patients with degenerative lumbar spinal stenosis (DLSS) were included. They were subjected to a test protocol before and after surgery. The protocol consisted of two parts, a dynamic and an isometric Ito loading with a time frame of 60 s and accompanying rest of 120 s. The Ito test was repeated three times. EMG was measured bilaterally at the L4 level and L2 and was recorded using surface electrodes and collected (Biopac Systems Inc.). EMG signal was expressed as RMS and median frequency (MF). Muscle tissue oxygen saturation (MrSO_2_) was monitored using a near-infrared spectroscopy (NIRS) device (*INVOS*® 5100C Oxymeter). Two NIRS sensors were positioned bilaterally at the L4 level.

The intensity of the leg and back pain and perceived exertion before, during, and after the test was evaluated with a visual analogue scale (VAS) and Borg RPE-scale, respectively.

**Results:**

All patients were able to perform and complete the test protocol pre- and postoperatively. A consistency of lower median and range values was noted in the sensors of EMG1 (15.3 μV, range 4.5–30.7 μV) and EMG2 (13.6 μV, range 4.0–46.5 μV) that were positioned lateral to NIRS sensors at L4 compared with EMG3 (18.9 μV, range 6.5–50.0 μV) and EMG4 (20.4 μV, range 7.5–49.0 μV) at L2. Right and left side of the erector spinae exhibited a similar electrical activity behaviour over time during Ito test (60 s). Regional MrSO_2_ decreased over time during loading and returned to the baseline level during recovery on both left and right side. Both low back and leg pain was significantly reduced postoperatively.

**Conclusion:**

Simultaneous measurement of surface EMG and NIRS seems to be a promising tool for objective assessment of paraspinal muscle function in terms of muscular activity and local muscle oxygenation changes in response to isometric trunk extension in patients that have undergone laminectomy for spinal stenosis.

## Introduction

Laminectomy or decompression surgery for degenerative lumbar spinal stenosis (DLSS) is a procedure with proven patient benefits. This surgery has several surgical methods. However, the effects and/or the outcomes of these different methods have never been fully investigated or clarified [1–3]. The literature indicates there is a lack of proper tools to determine how the spinal muscles perform their task after an intervention, such as lumbar spinal surgery for DLSS and how it relates to experienced pain or other factors that have an impact of daily life and quality of life.

Qualitative and quantitative research protocols have been proposed and used either in isolation or in combination for *functional assessment* of the lumbar *spine*. Subjective assessment has been shown to be limited in determining the muscle functional status and therefore needs other objective methods to be considered. Measuring muscle electric activity with electromyography (EMG) has been shown to be a reliable method and has previously been used in studies for evaluating the functionality of muscles in the lumbar spine [4–11].

The supply of oxygen and nutrients and the exchange of deposits are key points in maintaining efficient muscle function. Previous studies have shown that in the lumbar spine muscle oxygenation decreased during static contractions using near-infrared spectroscopy (NIRS) that enables non-invasive, real-time monitoring changes in muscle oxygenation.

The lumbar spine muscles have extensively been investigated, but few studies addressed the muscle activity and oxygenation simultaneously, particularly in clinical practice [12–14]. To the best of our knowledge, hemodynamic and electrophysiological studies on patients that have previously undergone lumbar spine surgery are limited.

The purpose of this study is to develop and evaluate objective measurements utilizing EMG and NIRS in order to quantify the muscle functional status during isometric trunk extension pre- and postoperatively in a cohort of a limited number of patients operated with laminectomy for symptomatic lumbar spinal stenosis.

## Materials and methods

### Patient demographics

Twelve patients were investigated: eight women and four men; mean age 67 (range 52–9) years; mean body height 176 (163–184) cm; and mean body mass index 26 (22–28) kg/m2. The patients were referred by general practitioners due to confirmed symptomatic DLSS on magnetic resonance imaging (MRI).

### Inclusion and exclusion criteria

Patients with symptom duration of neurogenic intermittent claudication (NIC) 6 months or more that was confirmed by MRI were included in this study. The exclusion criteria were spinal fractures, disc herniation causing sciatica, infection, previous lumbar spinal surgery and degenerative spondylolisthesis greater than grade 1, and cauda equine syndrome.

The present study was approved by the Regional Ethical Review Board.

### Surgical procedure

All patients were operated with open laminectomy either with osteotomy or with resections of the spinal processes.

### Test protocol

**Fig. 1:**
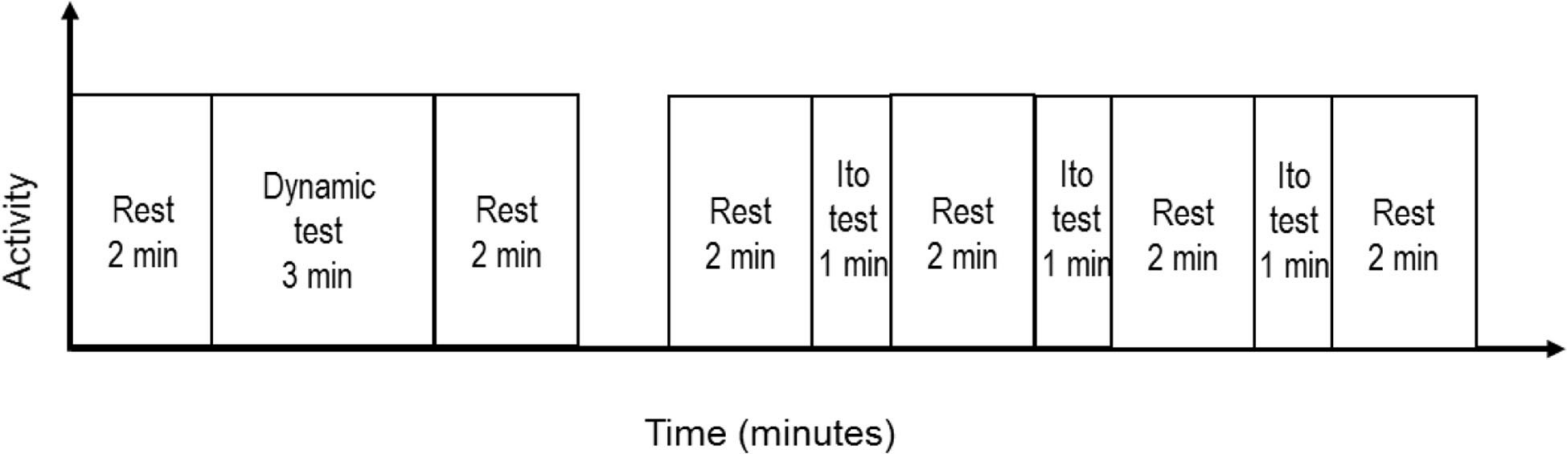
A schematic flowchart of the test protocol

Each patient performed identical dynamic trunk flexion–extension movements (dynamic test) and isometric trunk extensions (Ito test) pre- and 3 months postoperatively. Both left and right sides of paraspinal muscle activity and intramuscular oxygenation were simultaneously measured before, during, and after both the dynamic test and the Ito tests using EMG and NIRS. See Fig. 1 for a schematic flowchart of the test protocol.

### Dynamic trunk flexion–extension test

**Fig. 2:**
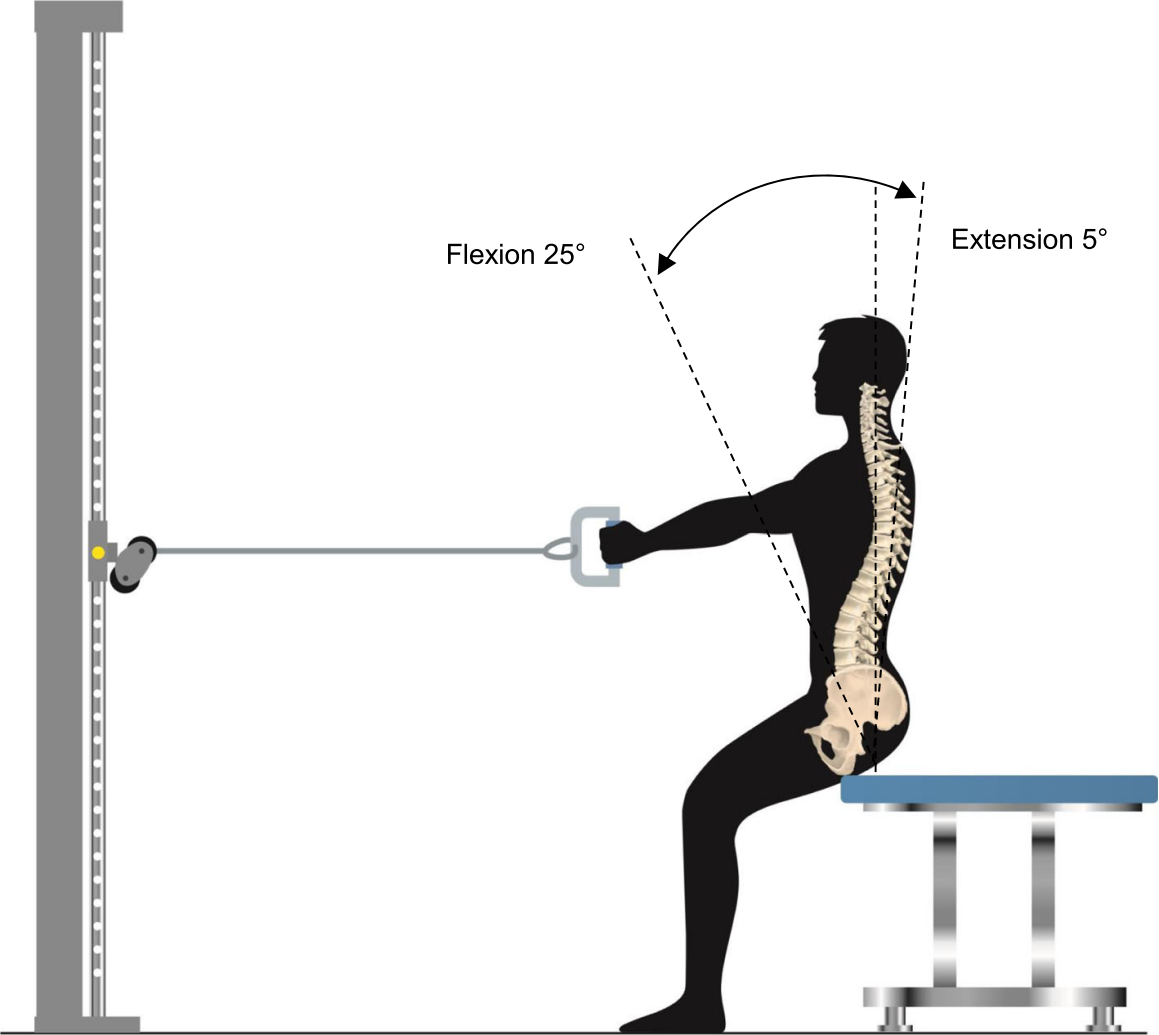
Dynamic trunk flexion–extension test. A full stroke from 25° flexion to 5° extension was repeated for 3 min at a rate of one per 4 s

All patients were instructed to sit in a semi-sitting position with 70° of hip flexion. They were then asked to lift and lower a weight (7 kg) using a Lojer pulley 14/20 traction device, from the floor to a specific height (Fig. 2) individualized according to the patient’s spinal length in order to obtain a lumbar movement amplitude of 25° flexion to 5° extension [15]. The test started with a trial for recognition, followed by 2 min rest for baseline data. The patients were instructed to keep their arms straight and keep a cyclic pace when performing the flexion–extension movement until fatigue or a maximum of 3 min (Fig. 2).

### Isometric trunk extension (Ito test)

**Fig. 3:**
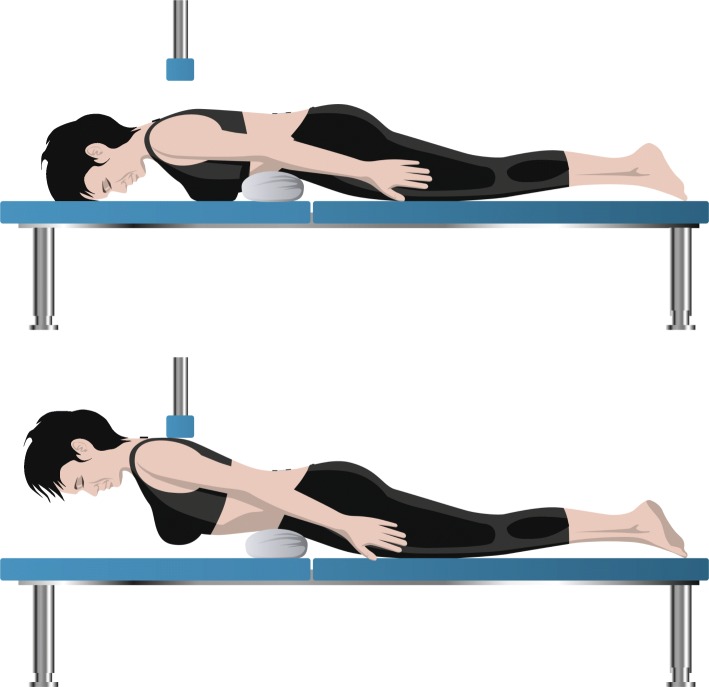
**a** Ito test starting position. **b** Isometric trunk extension (Ito test), active position held for max 1 min

The Ito test was performed according to Ito et al. with the patients in a prone starting position on an examination table [16]. A 10-cm-high pad was placed under the lower abdomen to decrease the lumbar lordosis. The arms were kept parallel to the body axis (Fig. 3a). The patients were instructed to lift their upper body off the examination table to an individual adjusted endpoint. The cervical spine was held in a neutral position, looking down, and both feet remained on the examination table throughout the entire test until voluntary fatigue or for a maximum of 1 min (Fig. 3b). The test started with a trial for recognition, followed by 2 min rest for baseline data and then three recorded trials with 2 min rest in between.

### Assessment of paraspinal muscle activity by EMG

**Fig. 4:**
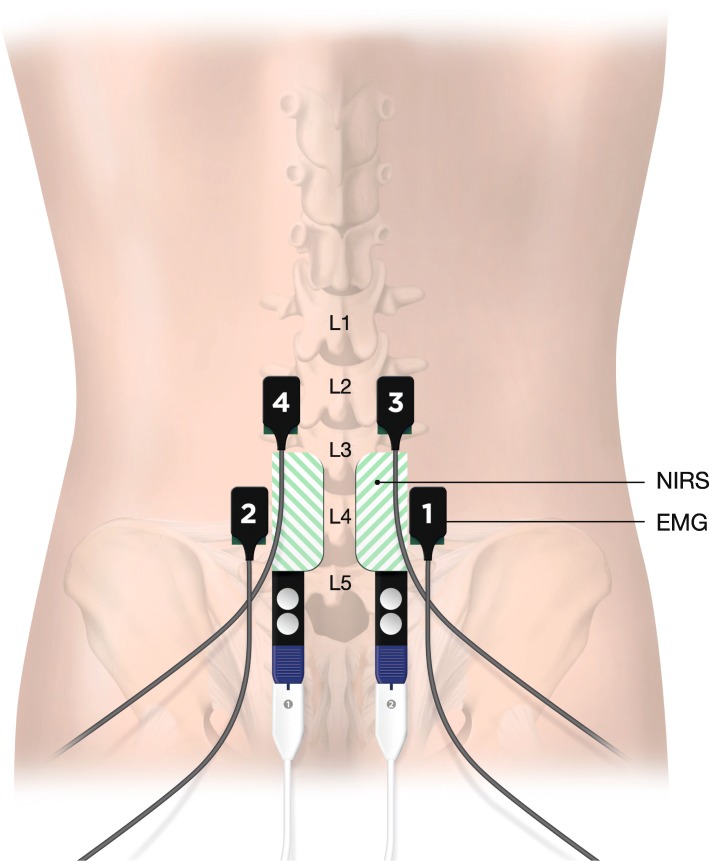
NIRS sensors 1–2 and EMG sensors 1–2 positioned at L4 level. EMG sensors 3–4 positioned at L2

To evaluate lumbar spine muscle activity, all patients were subjected to an EMG examination. Four Bagnoli single differential surface EMG electrodes (DE-2.1, Delsys Inc., USA) were placed bilaterally over the paraspinal lumbar muscles at L2–L4 level according to the protocol recommended by SENIAM (www.seniam.org) for evaluation of trunk or lower back muscle EMG activity (Fig. 4). EMG signals were amplified using a Bagnoli-16 unit which has a filter bandwidth of 20 to 450 Hz. Gain was set to 10 k. Signals were then transferred through the BNC output connections to a Biopac MP100 (Biopac Systems Inc., USA) using a Universal interface module (UIM100, Biopac Systems Inc., USA). Acqknowledge software by Biopac was used for collecting data and evaluation of RMS. EMG signals were sampled at 1000 Hz.

The root-mean-square (RMS) values of the EMG signal were calculated at time event = 0, 5, 10, 20, 30, 40, 50, and 60 s. A time window frame of 2 s was used to calculate a mean value at respective time event. EMG RMS values are thus presented as a function of time and merged with oxygen saturation data.

Median frequency (MF) was calculated using a 2-s time window in the 5–60 s of the Ito test loading time frame. A regression coefficient, referred as the slope, was then calculated for each Ito loading using a linear approximation.

### Assessment of paraspinal muscle oxygenation by NIRS

Localized changes in the paraspinal muscle tissue oxygen saturation (MrSO_2_) were monitored continuously during the dynamic test and the Ito test using a NIRS device (*INVOS*® 5100C Oxymeter, Somanetics, Troy, MI, USA*)*. Two self-adhesive disposable NIRS sensors (Adult SomaSensor® SAFB-SM, Somanetics, Troy, MI, USA) were positioned bilaterally over the paraspinal muscles 2 cm to the left and right of fourth lumbar vertebra (Fig. 4). The INVOS uses two wavelengths between 730 and 810 nm and two detectors with a centre separation from the light source of 30 and 40 mm, respectively. The penetration depth of the INVOS device is roughly 20–25 mm. For each patient, the MrSO_2_ values were determined every 5 s and collected.

### Pain evaluation

The intensity of low back pain (LBP) and leg pain were evaluated with a 10-cm visual analogue scale (VAS) ranging from 0 (no pain) to 10 (worst imaginable pain), before and after the dynamic test and the Ito test, respectively. The patients rated their perceived exertion with Borg RPE-scale [17], ranging from 6 (very light) to 20 (maximal exertion) after every test phase.

### Subcutaneous tissue thickness measurements

The skin and subcutaneous thickness at the paraspinal muscles at L4–5 was identified and measured using ultrasound (L10–5, Acuson CV70, Siemens Medical Solutions Inc., USA). Blood pressure was measured in the beginning and at the end of the test protocol using a pressure manometer (NAIS, Matsushita, Electronic Works, Japan).

### Data analysis

Data are presented as means and standard deviation (SD) unless otherwise indicated. A paired sample *t* test was used to compare data between preoperative and postoperative for each time point measured.

For both EMG and NIRS measurements, recorded values at *t* = 5 s are used as a normalizing value and all values recorded at *t* = 10, 20, 30, 40, 50 or 60s are expressed as a percentage of the value at *t* = 5 s. This procedure is used in the succeeding report of data evaluation unless noted. Statistical significance for all tests was accepted at the 5% level. The *Wilcoxon signed rank test was used* for comparison of pre- and postoperative VAS and Pearson’s Chi-Square test was used to compare pre- and postoperative RPE. Statistical analysis was conducted with IBM SPSS Statistics for Windows, Version 25.0. Armonk, NY: IBM Corp and Microsoft Excel (2016).

## Results

### Test protocol

All patients were able to perform and complete the test protocol pre- and postoperatively. The time expired between operation and the postoperative test session varied between 84 to 246 days with a median of 108 days.

### Assessment of paraspinal muscle activity by EMG

**Fig. 5:**
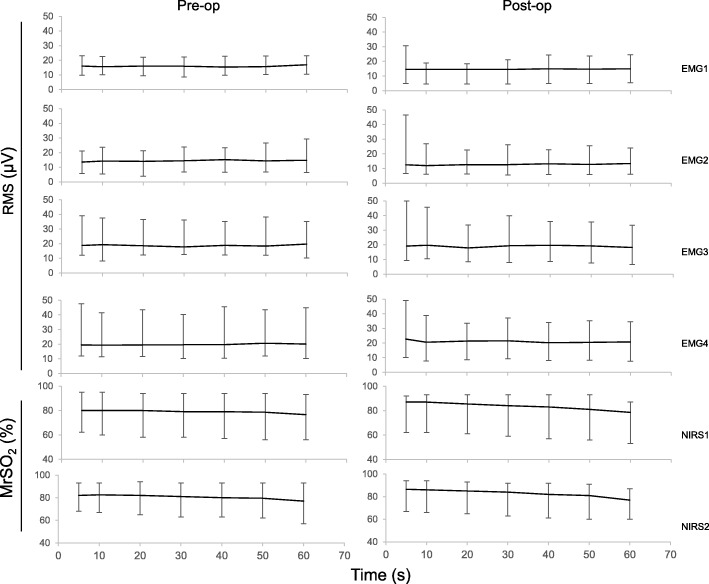
A summary of all collected data as a function of time. EMG (μV) and MrSO_2_ (%) values presented as median and range, based on all three Ito tests merged for respective sensor

**Fig. 6:**
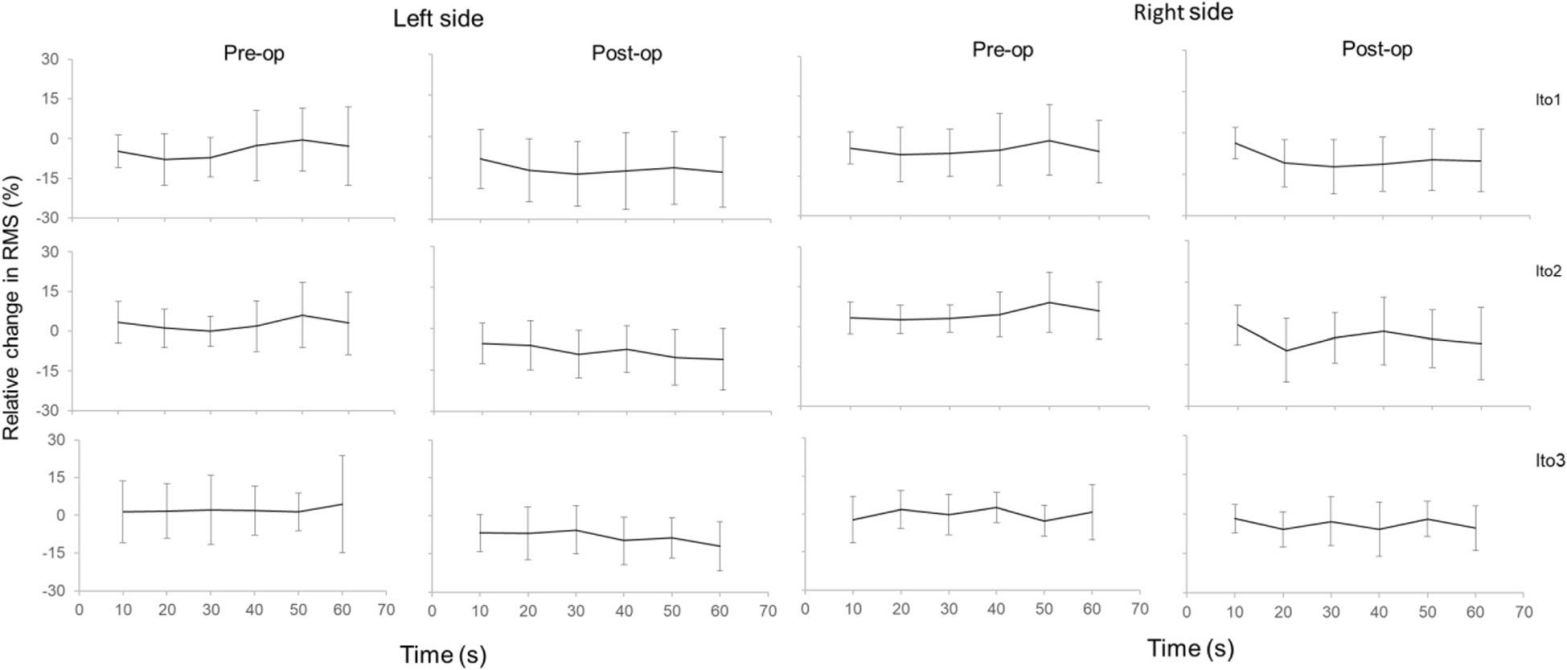
Relative change in RMS expressed as percent over time. Left (EMG4) and right side (EMG3), pre- and postoperatively. All three loading blocks, Ito 1, 2, and 3. Values presented are mean, error bars 95% CI

A consistency of lower median and range values was noted in the sensors of EMG1 (15.3 μV, range 4.5–30.7 μV) and EMG2 (13.6 μV, range 4.0–46.5 μV) compared with EMG3 (18.9 μV, range 6.5–50.0 μV) and EMG4 (20.4 μV, range 7.5–49.0 μV). Data is presented and shown in Fig. 5. The right and left side of the erector spinae exhibited a similar behaviour and did show a significant difference at time event *t* = 20, 30, 50, and 60s in Ito test 2, between pre- and postoperative values. This was not shown in Ito test 1 or 3. There was no difference in RMS percentage values of the EMG signal between left and right side muscular activity pre- or postoperatively (Fig. 6). The time-dependent behaviour (0–60 s) of the EMG measurements differed slightly between pre- and postoperatively. Preoperatively, the values in Ito test 1 decreased as a function of time, whereas in Ito test 2 they increased and in Ito 3 a more independent behaviour was noted. Postoperatively, a more consistent behaviour was noted where all values did decrease over time in all Ito tests. Median frequency displayed lower values postoperative compared with preoperative in both sensors and in all Ito tests as can be seen in Table 1.

**Table 1:** Median frequency from EMG3 and EMG4, presented as mean value and standard deviation based on all subjects

EMG sensor	Ito	Pre	SD.	Post	SD
		Mean value (Hz)		Mean value (Hz)	
# 3	Ito 1	67.6	13.9	64.3	10.7
	Ito 2	70.4	13.9	65.2	9.5
	Ito 3	71.0	14.1	64.0	12.7
# 4	Ito 1	66.9	12.4	60.8	11.2
	Ito 2	69.7	11.9	62.6	10.3
	Ito 3	68.3	12.7	62.3	12.1

### Assessment of paraspinal muscle oxygenation by NIRS

Regional muscle oxygenation as recorded by NIRS showed great similarities between left and right side sensors. Left side sensor (NIRS1) yielded a mean value of 79% and a range of 53 to 95%, whereas right side sensor (NIRS2) yielded a mean value of 80% and a range of 57 to 94%.

**Fig. 7:**
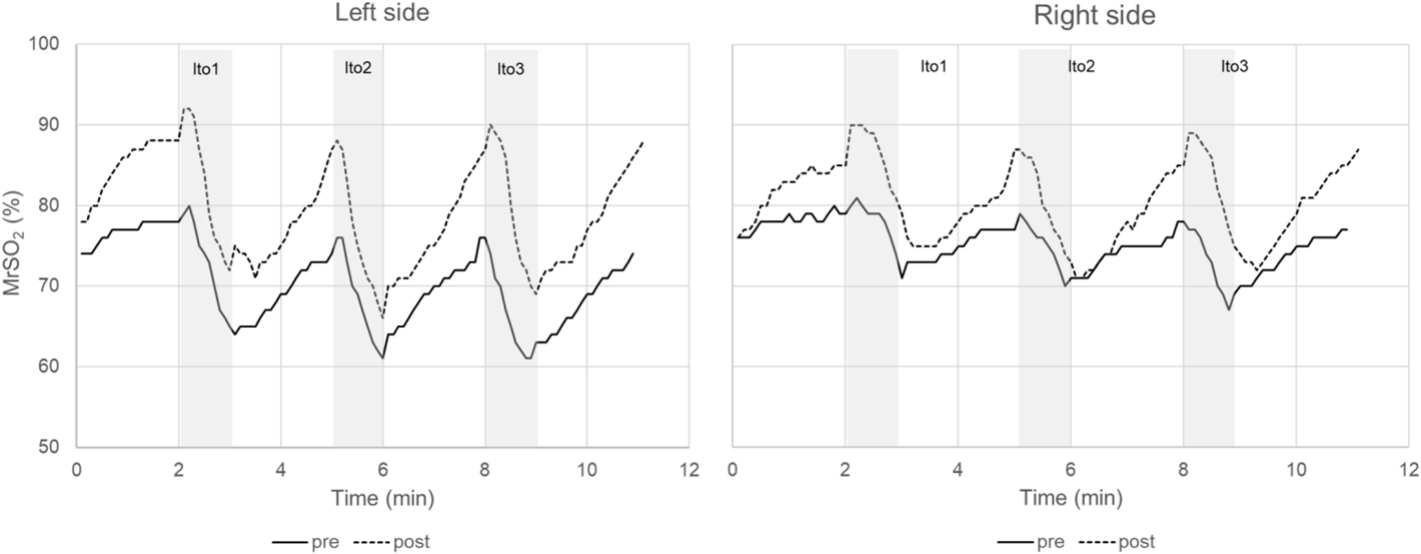
Original paraspinal muscle regional tissue oxygen saturation (MrSO_2_) trends of the left and right sides before, during, and after the Ito tests in a representative patient

The pre- and postoperative MrSO_2_ trends of the left and right paraspinal muscles over time before, during, and after the Ito tests in a representative patient are shown in Fig. 7.

**Fig. 8:**
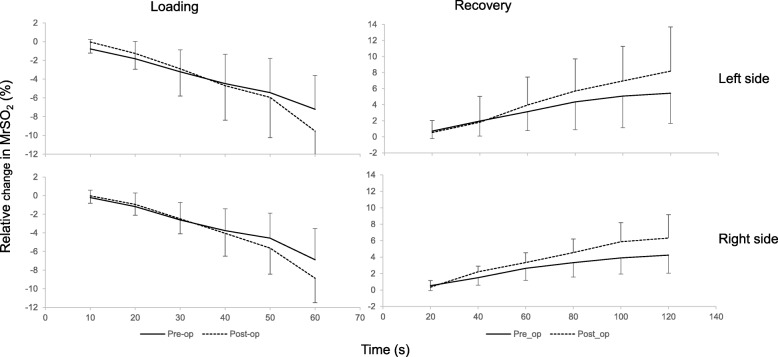
Relative changes of the paraspinal muscle regional tissue oxygen saturation (MrSO_2_) during and after the Ito test (*n* = 12). The MrSO_2_ values (mean ± SD) are expressed as % of baseline value (value at 5 s)

During loading, the mean (average of the three Ito tests) relative change in pre- and postoperative MrSO_2_ for both left and right paraspinal muscles are presented in Fig. 8. Both pre- and postoperative MrSO_2_ decreased over the Ito test compared to the baseline value at *t* = 5 s and the MrSO_2_ trends appeared similar between sides. Although postoperative MrSO_2_ value was lower than preoperative values at the end of the Ito test (60 s) for both sides, no significant differences were observed. During recovery at the cessation of the Ito test, both pre- and postoperative MrSO_2_ increased compared to the baseline value and it showed a similar trend for both sides as shown in Fig. 8. Although the postoperative MrSO_2_ value was higher than preoperative value at the end of recovery for both sides, no significant differences were observed.

### Pain evaluation

Pain was reduced postoperatively as implied by the significant difference between pre- and postoperative observations of VAS in the back, both before (*p* = 0.017) and after (*p* = 0.007) Ito tests, as well as of VAS in the leg after the Ito tests (*p* = 0.043) (Table 2). No difference was noted in perceived exertion between pre- and postoperative parameters (Table 2). Before operation, the patients indicated greater pain in the back and leg after performing the Ito tests, compared to before the tests, though this was not significant. After operation, the patients did not report more pain in the back and leg after performing the Ito tests.

**Table 2:** VAS and RPE before and after all three Ito tests, pre- versus postoperatively

	Ito test	Pre-op	Post-op	*P* values
VAS back	Before test 1	45 (0:70)	0 (0:18)	*p* = 0.017^1^
	After test 3	50 (0:70)	0 (0:18)	*p* = 0.007^1^
VAS leg	Before test 1	0 (0:42)	0 (0:0)	ns^1^
	After test 3	26 (0:57)	0 (0:0)	*p* = 0.043^1^
RPE	After test 1	14 (13:17)	13 (11:15)	ns^2^
RPE	After test 2	14 (13:17)	13 (11:17)	ns^2^
RPE	After test 3	15 (13:17)	13 (11:17)	ns^2^

Values are presented in median (Q25:Q75).

^1^Wilcoxon signed rank test, pre- vs. postoperative VAS (visual analogue scale)

^2^Chi^2^-test, pre- vs. postoperative RPE (the Borg rating of perceived exertion)

### Thickness of skin and subcutaneous tissue

Subcutaneous tissue thickness measurements displayed no difference between right side, median 10.5 mm (range 8.7–20.0 mm), and left side, median 11.2 mm (range 8.6–20.0 mm).

## Discussion

The main findings in the present study highlights that all patients could perform the experimental protocol both pre- and postoperatively. Furthermore, both EMG and NIRS methods were shown to be able to measure the paraspinal muscle activity and changes of oxygenation in response to isometric trunk extensions in patients pre- and postoperatively.

### Experimental protocol

The dynamic trunk flexion–extension test was considered mainly as a warm up for the test subject. The isometric Ito test applied in our experimental protocol was easy to perform, safe, and comfortable for both pre- and postoperative patients. Loading time was set to 60 s which is well within the endurance time values that Ito et al. reported for female (mean 70.1 s) and male (mean 85.1 s) with chronic low back pain (CLBP) [16]. The majority of the patients were thus believed to be able to perform the test without reaching their endurance limit. However, *this finding is not in line* with the findings of Demoulin et al. (2008), who showed that the Ito test was less comfortable and more difficult to standardize. The Biering–Sorenser test is often suggested for measuring trunk extensor muscles, particularly in endurance tests when fatigue of the muscles is investigated. The Ito test share the same principles but use greater trunk support and in doing so, become more user friendly in a cohort of LBP patients that might exhibit fear of movement.

### General observations

#### EMG

Muscle activity as recorded by EMG and presented as RMS in all 4 measuring sites displayed an activity level within the same range as previous studies, using similar loading mode and protocols [18, 19]. In general, a strong valid EMG recording was possible from all sensors, but highly dependent on a good reference position (C7) as well as its conductivity. The RMS values recorded varied were dependent on location of sensor and independent of pre- or postoperative measuring session or Ito test. EMG sensor 1 and 2 were placed more lateral and at a lower spinal level than sensor 3 and 4, thereby recording different anatomical structures. The level of muscle activity is also specific to the loading mode as reported by Tucker et al. [20]. The Ito test used in this study is viewed mainly as an upper body extension movement without any intentional lateral bending or rotation.

#### NIRS

Muscle oxygenation responses in the paraspinal muscles showed a rapid decline during the Ito test and an increase toward the baseline value during recovery from the Ito test (Fig. 8). These results indicate an acute imbalance between oxygen supply and oxygen demand in the working muscles during the Ito tests [21]. Furthermore, increased intramuscular pressure during muscle contractions reduces muscle blood supply and oxygen delivery to the active muscles [22]. The rapid increase in muscle oxygenation during the immediate post-exercise period counteracts the reduced oxygen supply due to muscle contractions. The muscle oxygenation trends observed in this study were similar for the pre- and postoperative patients across both right and left sides, and our results are consistent with previous studies [12, 23]. There were *large variations shown for muscle oxygenation levels among patients* because the level of change may reflect a variation in work intensity and may also be influenced by differences in the location of their pain.

#### Clinical outcome

Since the primary objective of the present study was to propose and validate a protocol suitable for evaluation of lumbar muscle functionality during isometric loading, the cohort was limited in terms of numbers of patients. This has an adverse effect on the possibility to draw any statistical significant conclusions from the material. However, trends or associations could be identified and the cohort can provide information for a future power analysis.

#### Left–right symmetry

Muscle activity as recorded by EMG and oxygenation are plausible properties that would be able to detect differences in left–right symmetry. No significant EMG difference was shown to occur in observed patients in neither the pre- nor postoperative measurements between left and right side of the lumbar spine in any load block. However, a weak trend was noted in the postoperative group at time event *t* = 30, 40, 50, and 60 in test load block 2 and 3.

#### Pre–postoperative differences

In the present study, the time-dependent behaviour of the muscle activity as recorded by EMG exhibited pre- and postoperative differences. The postoperative EMG data displayed a more uniform expression since in all Ito tests the values decreased as a function of time. Preoperatively, a more inconsistent behaviour was present; in test 1 the values decreased, but in test 2 and 3 the values increased as a function of time. This could speculatively be an expression of fatigue, namely that the RMS may increase over time. A permanent change in spinal muscular morphometry and substance composition has been observed in patients postoperatively [24–27].. MRI and other image generating tools seem to be the dominating means to evaluate such changes, but EMG offers the opportunity to evaluate the functionality of the muscles and particularly time- and load-dependent properties such as fatigue.

#### Fatigue

The moderate isometric load level reached in a Ito test may not be sufficient to initiate a marked fatigue development within 60 s and fatigue is therefore not as well detectable as it would with a load level in the vicinity of maximal voluntary contraction (MVC). Changes in MF and RMS values of the EMG signal as a function of time during isometric loading has been previously suggested as being a reflection of muscle fatigue [7, 10]. However, the observed phenomenon that MF decrease and RMS increase as a function of time appears not to be consistent in the literature. Plausible causes for this could be low load levels, short loading time events or that the investigated muscles exhibit an unknown behaviour, such as pato-physiological behaviour should not be ruled out.

In the present study, the cohort where spinal patients diagnosed with spinal stenosis. They are subjected to a fairly low load compared to the MVC but are still within the ranges that an individual may be subjected to during daily life, i.e., 20–40% of MVC [28]. A notable difference between pre- and postoperative MF and RMS inter-relationship was present.

### Normalization of data

In order to evaluate individual properties and compare between individuals a normalizing of data is proposed. The value at 5 s is used as a normalizing reference value for all parameters in order to determine a relative expression in percentage as a function of time. The main focus is thereby shifted to the time-dependent properties of the parameters.

### Limitations

#### Sensor placement

Sensors EMG 1 and EMG 2 were placed lateral to the NIRS sensors, this means that the muscle activity recorded was from a slightly different muscle bulk than the EMG 3 and EMG 4 sensors. A consistently lower RMS value was recorded for EMG 1 and EMG 2 as compared to EMG 3 and EMG 4 throughout the study, indicating a reduction in lateral muscle activity compared with the more centralized positioning of the sensors. This finding has previously been shown by others and is attributed to the anatomy of the erector spinae and multifidus muscle [29, 30]. The EMG 1 and EMG 2 sensors appeared to be more consistent in picking up electrical environment noise than EMG 3 and EMG 4. Consequently, more of their recordings were rejected in the analysis. Based on these two major findings, it was elected to remove these from the final analysis and propose only the use of EMG 3 and EMG 4 sensors in the future studies.

#### Thickness of the skin and subcutaneous tissue

The thickness of the skin and subcutaneous tissue ranged from 8.6 to 20 mm in the present study. We found no correlations between the thickness of the skin and subcutaneous tissue and MrSO_2_ values.

#### Technical issues

The location of EMG sensors was defined and dependent on where the NIRS sensors were placed with reference to the dorsal midline of the subject. Previous test–retest studies of EMG sensors placement yield a signal variation expressed as ICC on the order of 0.52–0.91 which is to be considered when comparisons between different test occasions are made [8, 31].

Electrical environmental noise was present mainly on the EMG 1 and EMG 2 sensors which were located lateral to the NIRS sensors and often occurred on slightly high BMI subjects where speculatively the muscle fat ratio is low and more susceptible to electrical interference, therefore creating a poor signal to noise ratio. Quality control of EMG was performed on data derived from the initial resting phase to assure a minimum of electrical noise superimposed on the true muscle signal. Data containing 50 Hz noise were omitted from the analysis as noted.

## Conclusion

The present study suggests that simultaneous measurements of surface EMG and NIRS is a promising tool for objective assessment of paraspinal muscle function in terms of muscular activity and local muscle oxygenation changes in response to isometric trunk extensions in both pre- and postoperative analysis of patients that have undergone laminectomy for spinal stenosis.

## Data Availability

The datasets used and/or analysed during the current study are available from the corresponding author on reasonable request.

## Abbreviations

EMG: Electromyography
NIRS: Near-infrared spectroscopy
DLSS: Degenerative lumbosacral stenosis
NIC: Neurogenic intermittent claudication
RMS: Root-mean-square
MF: Median frequency
MrSO_2_: Muscle tissue oxygen saturation
LBP: Low back pain
VAS: Visual analogue scale
RPE: Rating of Perceived Exertion
MRI: Magnetic resonance imaging
ITO test: Isometric trunk extension test
